# RhD Specific Antibodies Are Not Detectable in HLA-DRB1^*^1501 Mice Challenged with Human RhD Positive Erythrocytes

**DOI:** 10.1155/2014/470242

**Published:** 2014-12-31

**Authors:** Lidice Bernardo, Gregory A. Denomme, Kunjlata Shah, Alan H. Lazarus

**Affiliations:** ^1^The Canadian Blood Services, Canada; ^2^Department of Laboratory Medicine and the Keenan Research Centre in the Li Ka Shing Knowledge Institute of St. Michael's Hospital, 30 Bond Street, Toronto, ON, Canada M5B 1W8; ^3^Immunohematology Reference Laboratory, BloodCenter of Wisconsin, Milwaukee, WI 53226, USA; ^4^Department of Transfusion Medicine, St. Michael's Hospital, Toronto, ON, Canada M5B 1W8; ^5^Departments of Medicine and Laboratory Medicine & Pathobiology, University of Toronto, Toronto, ON, Canada

## Abstract

The ability to study the immune response to the RhD antigen in the prevention of hemolytic disease of the fetus and newborn has been hampered by the lack of a mouse model of RhD immunization. However, the ability of transgenic mice expressing human HLA DRB1^*^1501 to respond to immunization with purified RhD has allowed this question to be revisited. In this work we aimed at inducing anti-RhD antibodies by administering human RhD^+^ RBCs to mice transgenic for the human HLA DRB1^*^1501 as well as to several standard inbred and outbred laboratory strains including C57BL/6, DBA1/J, CFW(SW), CD1(ICR), and NSA(CF-1). DRB1^*^1501 mice were additionally immunized with putative extracellular immunogenic RhD peptides. DRB1^*^1501 mice immunized with RhD^+^ erythrocytes developed an erythrocyte-reactive antibody response. Antibodies specific for RhD could not however be detected by flow cytometry. Despite this, DRB1^*^1501 mice were capable of recognizing immunogenic sequences of Rh as injection with Rh peptides induced antibodies reactive with RhD sequences, consistent with the presence of B cell repertoires capable of recognizing RhD. We conclude that while HLA DRB1^*^1501 transgenic mice may have the capability of responding to immunogenic sequences within RhD, an immune response to human RBC expressing RhD is not directly observed.

## 1. Introduction

The RhD antigen is a clinically important human blood group that can be a primary target in hemolytic disease of the fetus and newborn (HDFN) as well as some cases of autoimmune hemolytic anemia. Antibodies to RhD (anti-D) have been used for many years to prevent HDFN. The ability to manipulate and study the immune response to the RhD antigen in the prevention of HDFN has been hampered by the lack of a murine model to study this antigen. Although never formally published, it has been generally considered that standard laboratory mice do not make an immune response to the RhD antigen [1]. However, the more recent ability of creating transgenic mice expressing functional human HLA antigens has allowed this question to be revisited in a murine model.

The Aberdeen group has successfully induced an immune response to solubilized RhD protein in humanized mice that express the human HLA-DRB1^*^1501 allele [1]. Human HLA class II DR has been found as a major restricting element for human T-helper cells specific for RhD protein [2], and the HLA-DRB1^*^1501 allele is significantly overrepresented in RhD negative donors who have produced anti-RhD antibodies in response to RhD-positive RBCs [3]. In particular, the expression of the HLA DRB1^*^1501 transgene was found to confer on mice the ability to respond to immunization with purified RhD protein [1].

In addition to being able to stimulate an immune response, T cell epitopes derived from RhD protein sequences were also shown to induce oral tolerance to the RhD antigen in the HLA-DRB1^*^1501 murine model. While an immune response to purified RhD protein is of interest, the ability of an immune response to be generated to naturally expressed RhD on the surface of red cells is needed to move forward with relevant murine models. Thus far, an immune response to RhD expressed on the surface of erythrocytes in mice expressing HLA-DRB1^*^1501 has not yet been addressed.

In this work we aimed at inducing an anti-RhD antibody response by administering human RBCs expressing RhD in mice expressing HLA DRB1^*^1501 [4]. It is important to mention that the HLA DRB1^*^1501 mouse strain used here is different from the one used by Hall and his collaborators in 2005. Specifically, the HLA DRB1^*^1501 mice used in our study lack the expression of functional murine MHC class II, forcing the restricting element for immune responses through HLA DRB1^*^1501 [4]. In addition, conventional inbred and outbred mouse strains were also challenged with human RhD-positive RBCs to formally assess if standard strains of mice can generate anti-RhD specific antibody responses.

The results showed that when HLA DRB1^*^1501 transgenic mice are challenged with RhD positive RBC under a variety of conditions, despite the development of an immune response to the red cells, no antibodies to RhD were detected by flow cytometry. However, the results of the peptide studies were consistent with the presence of a B cell repertoire capable of recognizing each of the three immunogenic sequences evaluated. We conclude that while HLA DRB1^*^1501 transgenic mice have the capability of responding to sequences from RhD, an immune response specific for human RBC expressing RhD is not directly observed.

## 2. Materials and Methods

### 2.1. Mice

HLA-DRB1^*^1501 transgenic mice expressing the human HLA-DRB1^*^1501 allele, without endogenous class II molecules, were kindly provided by Dr. Chella David (Mayo Medical School, Rochester, MN, USA) [4]. The only functional class II molecules on DRB1^*^1501 antigen-presenting cells are the human class II molecules. C57BL/6 and DBA1/J mouse strains were purchased from Jackson Laboratory (Bar Harbor, ME. USA). Outbred mouse strains CFW(SW) and CD1(ICR) were purchased from Charles River (Montreal, QC, Canada) while NSA(CF-1) was bought from Harlan Sprague Dawley Inc. (Indianapolis, IN, USA). All mouse work was approved by the St Michael's Hospital animal care committee and mice were housed in the St Michael's Hospital Research Vivarium.

### 2.2. Immunization of Mice with Human Red Blood Cells

Whole blood and fully leukoreduced RBC units were obtained from The Canadian Blood Services Network Centre for Applied Development (NetCAD) and the work was approved by The Canadian Blood Services Research Ethics Board. Before immunization, RBCs taken from the units were washed three times in phosphate-buffered saline (PBS), pH 7.2, and counted using Guava EasyCyte Mini System cell analyzer (Guava Technologies, Hayward, CA, USA). Mice were challenged with one dose of 10^8^ human RhD positive RBCs (DRB1^*^1501*n* = 4, C57BL/6 *n* = 3, DBA1/J *n* = 2, CFW(SW) *n* = 3, CD1(ICR) *n* = 4, NSA(CF-1) *n* = 4). DRB1^*^1501 mice were additionally immunized with 10^8^ human RhD positive RBCs in the presence of CpG ODN adjuvant (Magic Mouse Adjuvant, Creative Diagnostics, NY, USA) (*n* = 2), or with two doses of 10^8^ human RhD positive RBCs administered 21 days apart without adjuvant (*n* = 2). Untreated mice were used as negative controls (*n* = 2). All the mice were bled for serum fifteen days after the first or seven days after the second challenge.

### 2.3. RhD Peptide Design and Immunization

**Figure 1:**
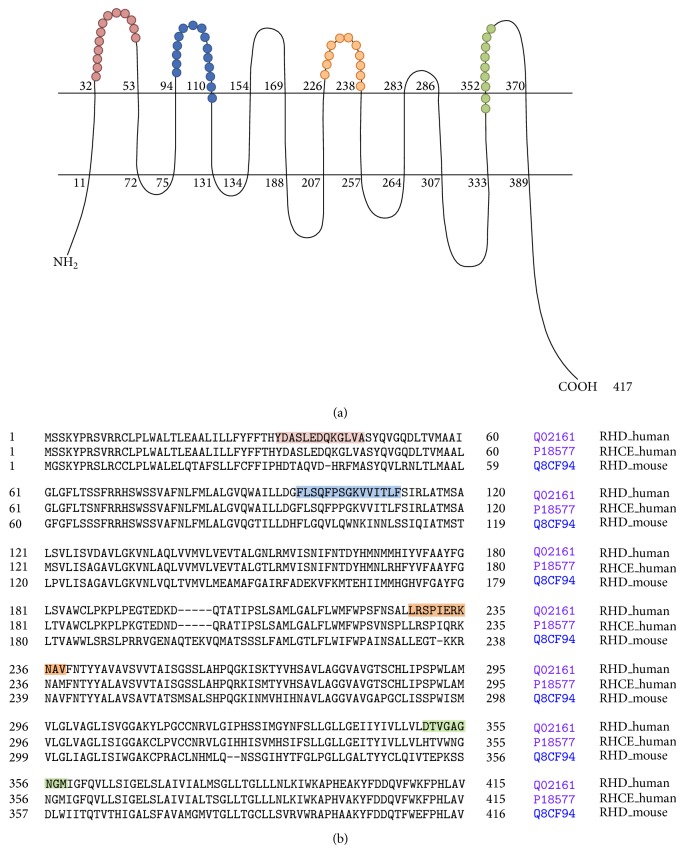
Predicted topographic features of the RhD protein (a) [5, 13] and alignment of human RHD, human RHCE, and mouse RHD sequences (b). Peptides 1–4 are highlighted in colors. Peptide 1: red; Peptide 2: orange; Peptide 3: green; Peptide 4: blue.

Peptides were designed and selected according to the human RhD sequence published in gene bank (accession number L08429). Putative linear epitopes were predicted using the antibody epitope prediction tool of the Immune Epitope Data Base (IEDB) Analysis Resource (http://tools.immuneepitope.org/tools/bcell/iedb_input). Four peptides that theoretically correspond to extracellular regions of the human RhD protein and that are different in sequence from mouse RhD [5] were selected (Table 1, Figure 1). Peptides were synthesized by Peptides International and shipped lyophilized (Louisville, Kentucky, USA). Peptides 1 to 3 were successfully solubilized in 1 M ammonium bicarbonate. Unfortunately, peptide 4 was not soluble in up to 10% organic solvent and was therefore not used. Peptides 1 to 3 were successfully linked to Keyhole Limpet Haemocyanin (KLH) (Sigma-Aldrich, St Louis, MO, USA) using a cysteine added at the C-terminus and Sulfo-SMCC (4-(N-Maleimidomethyl) cyclohexane-1-carboxylic acid 3-sulfo-N-hydroxysuccinimide ester sodium salt) (Sigma-Aldrich, St Louis, MO, USA) used as a cross-linker. The coupling efficiency of the cysteine containing peptides to KLH was determined by a cysteine assay using 5,5′-Dithiobis(2-nitrobenzoic acid) (DTNB or Ellman's reagent) which reacts with sulfhydryl groups at pH 8.0 to produce a chromophore with maximum absorption at 412 nm. The coupling efficiency was then calculated by measuring the concentration of the initial and residual cysteine-peptide in the assay mixture from a standard curve of cysteine (Sigma-Aldrich, St Louis, MO, USA). DRB1^*^1501 mice were separately immunized with three doses of 50 *μ*g of peptide 1, 2, or 3 coupled to KLH and emulsified in Freund's adjuvant (complete Freund's adjuvant for the first dose and incomplete for the second and third) (Sigma-Aldrich, St Louis, MO, USA), administered 14 days apart (2 mice per peptide). Mice were bled via the saphenous vein at the indicated times and serum was collected for detecting anti-peptide, anti-KLH, and anti-human RBC antibodies.

### 2.4. Analysis of the Antibody Response to RhD Peptides

To detect antibodies against the synthetic peptides themselves, ELISA plates (Cat # 07-200-35, Thermo Fisher Scientific Inc., MA, USA) were coated with 10 *μ*g/mL of the corresponding peptide in PBS. After overnight incubation at 4°C, plates were washed with 0.05% Tween 20 in PBS and blocked with 2% (wt/vol) bovine serum albumin (BSA) (Sigma-Aldrich, St Louis, MO, USA) in PBS for 2 hours at 22°C. After washing, serum samples (end point dilutions) were then added and incubated at 22°C for 1.5 hours. The plates were then washed and incubated with alkaline phosphatase F(ab′)_2_ fragment goat anti-mouse IgG, Fc*γ* fragment specific (Jackson Immunoresearch Laboratories, IN, USA). After 1 hour of incubation at 22°C, plates were washed again and 1 mg/mL p-nitrophenyl phosphate (Sigma-Aldrich, St Louis, MO, USA) in 0.001 mol/L MgCl_2_, 9.7% diethanolamine, pH 9.6, was added to the plates. Plates were read by an ELISA reader at 405 nm after 15 to 30 minutes. Antibody titers were defined as the highest serum dilution that showed a positive value.

### 2.5. Analysis of the RBC-Specific Antibody Response

Sera from mice challenged with human RhD positive RBC were tested for antibodies using RhD positive and RhD negative RBC by flow cytometry [6]. RBCs were washed three times in PBS, pH 7.2, and 2 × 10^6^ cells incubated with 20 *μ*L of serum diluted to 1/100 at 22°C for 1 hour, followed by 2.5 *μ*g/mL goat F(ab′)_2_ anti-mouse IgG (FITC conjugated) before analyzing by a Guava EasyCyte Mini System cell analyzer. To selectively detect RhD specific antibodies, the sera were first adsorbed with RhD negative RBC followed by an assessment of binding to RhD positive RBC. Serum adsorptions were performed by incubating packed RhD negative RBC with sera at 22°C for 1 hour under shaking conditions. Tubes were then centrifuged and the supernatant containing the serum dilutions was collected. A second adsorption cycle was also done at 4°C. Adsorbed serum dilutions were assessed against human RhD positive RBC as above. Human polyclonal anti-D serum (WINRHO, Cangene bioPharma Inc., MD, USA) was used as a positive control.

## 3. Results

### 3.1. Response of Selected Standard Mice to Immunization with Human RhD Positive Red Blood Cells

**Figure 2:**
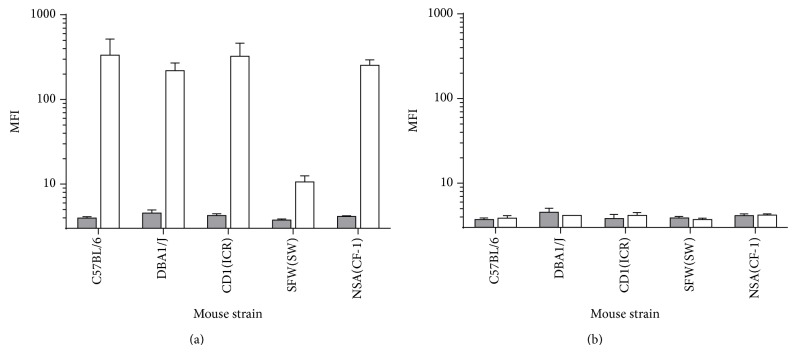
Immunization of standard laboratory mice with human RhD positive RBC induces RBC-reactive but not RhD-specific IgG as detected by flow cytometry. Conventional inbred and outbred mice were immunized with a single dose of 10^8^ RhD positive RBC (blood group A) (white bars) (C57BL/6 *n* = 3, DBA1/J *n* = 2, CFW(SW) *n* = 3, CD1(ICR) *n* = 4, NSA(CF-1) *n* = 4). Untreated mice were used as negative controls (grey bars) (*n* = 2). (a) Antibodies reactive with human RhD positive RBC (blood group A). (b) Antibody binding to RhD positive RBC after adsorbing the sera with human RhD^−^ cells (blood group A). MFI: mean fluorescence intensity.

Although it has been considered that standard laboratory mice do not make an immune response to the RhD antigen [1], there is no published data that we are aware of regarding the immune response to the RhD antigen when mice are challenged with human RBCs. To address this, some of the most commonly used inbred and outbred (Swiss and non-Swiss origin) mouse strains were challenged with human RhD positive RBCs and the antibodies reactive with human RBC as well as RhD specific antibodies were evaluated by flow cytometry. With the exception of SFW(SW), all the mouse strains tested [C57BL/6 (H2^b^), DBA/1J (H2^q^), CD1(ICR), and NSA(CF-1)] developed high levels of IgG antibodies reactive with human RhD positive RBC (Figure 2(a)). However, reactivity against human RhD positive RBC was not detected when sera were first adsorbed with RhD negative RBCs (Figure 2(b)), indicating that significant levels of RhD specific antibodies could not be detected.

The antibody response of SFW(SW) mice to human RBCs was particularly low. A potential explanation is that this strain contains a deletion in the promoter region of* H2-Ea* (which encodes the alpha chain of the MHC class II E*αβ* heterodimer), which strongly contributes to setting the ratio of CD4+ and CD8+ lymphocytes [7].

### 3.2. Immunization of HLA-DRB1^*^1501 Transgenic Mice with Human RhD Positive Red Blood Cells

**Figure 3:**
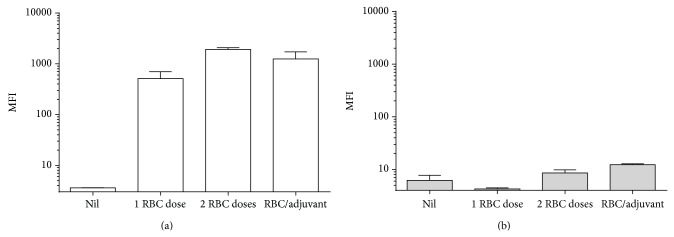
Immunization of DRB1^*^1501 mice with human RhD positive RBCs induces RBC-reactive but not RhD-specific IgG as detected by flow cytometry. DRB1^*^1501 mice were challenged with one (*n* = 4) or two doses of 10^8^ RhD positive RBC (*n* = 2) or 1 dose of 10^8^ RhD positive RBC emulsified in CpG ODN adjuvant (*n* = 2). Untreated mice were used as negative controls (Nil) (*n* = 2). (a) Detection of IgG reactive with human RhD positive RBC. (b) Detection of IgG reactive with human RhD positive RBC after adsorbing the sera with RhD negative cells. MFI: mean fluorescence intensity.

Considering that the expression of the HLA DRB1^*^1501 transgene was previously found to confer on mice the ability to respond to immunization with purified RhD protein [1], we examined HLA DRB1^*^1501 transgenic mice for an immune response by challenging these mice with human RhD positive RBC. Mice were challenged with one or two doses of 10^8^ human RhD positive RBC administered 21 days apart, or the same number of cells in the presence of the CpG ODN adjuvant. HLA-DRB1^*^1501 mice developed antibodies reactive with human RBC after challenge and the administration of two immunizations or the use of adjuvant increased the magnitude of the antibody response (Figure 3(a)). However, when sera produced from these mice were first adsorbed with RhD negative RBCs, antibodies specific for RhD could not be detected (Figure 3(b)).

### 3.3. Immunization of HLA-DRB1^*^1501 Transgenic Mice with Synthetic Peptides Corresponding to Human RhD Sequences

As in no case was there evidence that HLA-DRB1^*^1501 mice were capable of making a humoral immune response specific for the RhD antigen on the surface of red cells, we performed experiments with peptides from putative immunogenic regions of RhD to evaluate if these mice possess an appropriate B cell repertoire reactive with human RhD. Based on the predicted structure of the RhD protein on the RBC membrane (Figure 1), we synthesized four peptides that contain putative extracellular immunogenic regions of RhD. It is important to note that peptide 1 is identical between both Rh gene loci (RHD and RHCE) while peptides 2 and 3 displayed 82% and 67% sequence identity between RHD and RHCE, respectively. HLA-DRB1^*^1501 transgenic mice were then challenged with three doses of peptide 1, 2, or 3 conjugated to KLH. The fourth peptide was insoluble in buffers compatible for KLH conjugation and was not evaluated.

**Figure 4:**
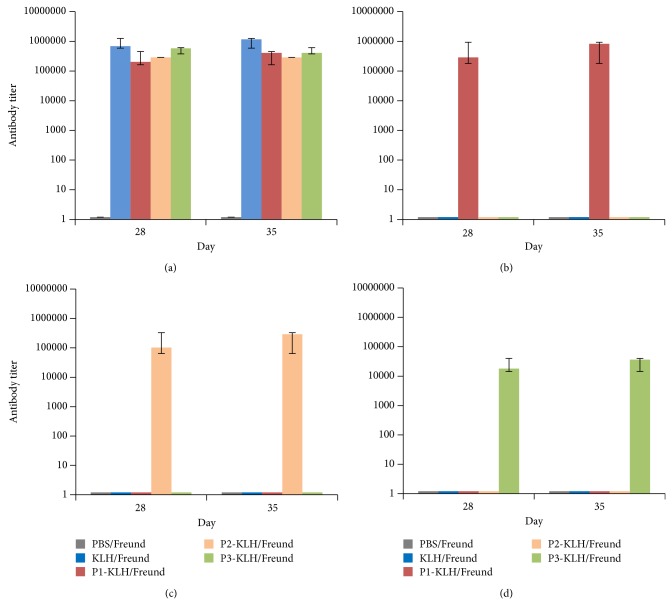
Immunization of DRB1^*^1501 mice with KLH-conjugated synthetic RhD peptides induces peptide specific IgG. Mice were immunized with each peptide (P) conjugated to KLH in Freund's adjuvant as indicated in Materials and Methods (2 mice per peptide). Detection of IgG specific for KLH (a), peptide 1 (b), peptide 2 (c), or peptide 3 (d) was assessed by ELISA. The sera tested were collected at days 28 and 35 (i.e., 14 days after 2nd and 7 days after the 3rd immunization, resp.).

Anti-peptide antibodies were successfully raised in HLA-DRB1^*^1501 mice from the sera collected 28 and 35 days after immunization; as detected by ELISA, using the immunizing peptides as antigens (Figure 4).

## 4. Discussion

Our studies provide evidence that selected inbred and outbred laboratory mice do not make an antibody response specific for naturally expressed human RhD. When we challenged standard inbred (H2^b^ and H2^q^) and outbred mice with human RhD positive RBC, the mice responded to the human RBC but antibodies specific for RhD protein were not observed from any of the mouse strains tested, despite using multiple RBC exposures or the addition of adjuvant to potentiate the immune response. Different incubation temperatures were also evaluated (data not shown). To the best of our knowledge, this is the first published study to show that selected conventional mice injected with human RBC do not develop antibodies specific to RhD protein detectable by flow cytometry.

An important consideration for the immunogenicity of the human RhD protein in mice is the degree of sequence homology with the mouse Rh protein. In the mouse, Rh protein is encoded by a single RH gene on chromosome 4 and exhibits only 60% sequence identity to the human proteins [8], which does not explain the lack of responsiveness to human Rh proteins. For instance, human RhD and RhCE are homologous proteins that have more than 90% sequence identity and still exposure to RhD can result in a potent immune response in a D-negative individual (RhC positive) [9–11].

A likely explanation could be that standard mice do not have the proper B or T cell repertoire to respond to the RhD protein. However, it has been previously demonstrated that mice transgenic for HLA DRB1^*^1501 respond to immunization with RhD purified protein [1], demonstrating that at least these mice (capable of expressing both human HLA-DRB1^*^1501 and murine MHC class II) have B cells specific for RhD epitopes. Conversely, when we challenged mice expressing only human HLA-DRB1^*^1501 with intact human RhD positive RBCs, antibodies specific to RhD were not observed though these mice successfully developed antibodies reactive with human RBCs. It could be possible that the naturally expressed human RhD protein is not a sufficiently dominant antigen in mice to generate a response. B or T cell lymphocyte responses are usually limited to a small proportion of the potential determinants on a protein antigen. Thus, when mice are challenged with human RBC expressing a variety of foreign proteins, the RhD protein could behave as a cryptic antigen. It is also likely that the antibody response to the RhD protein may be very low and that the flow cytometry assay is not sensitive enough to detect it. While flow cytometry has the advantage of measuring antibodies against the naturally expressed antigen on the red blood cell, it needs at least 100 molecules bound per cell to be detectable [12].

To confirm if mice expressing only HLA DRB1^*^1501 as a potential restricting element have the proper B cell repertoire to respond to human RhD sequences, these mice were challenged with RhD synthetic peptides. An anti-peptide specific antibody response was successfully induced after immunizing HLA DRB1^*^1501 mice with RhD synthetic peptides. These results are consistent with DRB1^*^1501 mice having a B and T cell repertoire able to recognize Rh immunogenic peptides and help demonstrate that the DRB1^*^1501 allele can theoretically be a restriction element for immune responses to RhD protein [1].

Although anti-D prophylaxis has been used to prevent HDFN in clinical medicine for more than four decades, the mechanism of action is still unclear. The lack of a mouse model of anti-D immunization has limited the study of HDFN to the RhD antigen as well as the protective mechanism of anti-D. However, naturally expressed human RhD proved to be poorly immunogenic in mice and, as a result, there remains a need for a relevant murine model.

A better understanding of the antigenic properties of RhD in mice will be helpful in designing future experiments to study the immune response to Rh. We have demonstrated herein that neither selected wild-type nor DRB1^*^1501 transgenic mice produce significant levels of anti-RhD specific antibodies in response to immunization with human RhD positive RBC, though these mice possess the B cell repertoire necessary for a response. The contribution of antigen dominance to RhD immunization may be a hurdle to overcome and would be a worthy next step to be addressed in detail.

## Figures and Tables

**Table 1:** Amino acid sequence and properties of the RhD peptides synthesized.

Peptide #	Amino acids	Sequence	M.W.	Sequence identity with RhCE	Net charge (pH 7)
1	34–46	YDASLEDQKGLVA**C** ^*^	1510.68	100%	−1
2	228–238	LRSPIERKNAV**C** ^*^	1384.66	82%	3
3	350–358	DTVGAGNGM**RR** **C** ^∗a^	1235.40	67%	2
4	97–111	FLSQFPSGKVVITLF**C** ^*^	1785.17	93%	2

^*^A cysteine was added at the C-terminus for conjugation purposes.

^a^Two arginines (R) were added to peptide 3 to increase its solubility.

## References

[1] Hall A. M., Cairns L. S., Altmann D. M., Barker R. N., Urbaniak S. J. (2005). Immune responses and tolerance to the RhD blood group protein in HLA-transgenic mice. *Blood*.

[2] Stott L.-M., Barker R. N., Urbaniak S. J. (2000). Identification of alloreactive T-cell epitopes on the Rhesus D protein. *Blood*.

[3] Urbaniak S. J. (2002). Alloimmunity to human red blood cell antigens. *Vox Sanguinis*.

[4] Khare M., Mangalam A., Rodriguez M., David C. S. (2005). HLA DR and DQ interaction in myelin oligodendrocyte glycoprotein-induced experimental autoimmune encephalomyelitis in HLA class II transgenic mice. *Journal of Neuroimmunology*.

[5] van Kim C. L., Mouro I., Cherif-Zahar B. (1992). Molecular cloning and primary structure of the human blood group RhD polypeptide. *Proceedings of the National Academy of Sciences of the United States of America*.

[6] Freedman J., Lazarus A. H. (1995). Applications of flow cytometry in transfusion medicine. *Transfusion Medicine Reviews*.

[7] Yalcin B., Nicod J., Bhomra A. (2010). Commercially available outbred mice for genome-wide association studies. *PLoS Genetics*.

[8] Liu Z., Huang C. H. (1999). The mouse Rhl1 and Rhag genes: Sequence, organization, expression, and chromosomal mapping. *Biochemical Genetics*.

[9] Westhoff C. M. (2007). The structure and function of the Rh antigen complex. *Seminars in Hematology*.

[10] Simsek S., de Jong C. A., Cuijpers H. T. (1994). Sequence analysis of cDNA derived from reticulocyte mRNAs coding for Rh polypeptides and demonstration of E/e and C/c polymorphisms. *Vox Sanguinis*.

[11] Flegel W. A. (2006). Molecular genetics of RH and its clinical application. *Transfusion Clinique et Biologique*.

[12] Zola H. (2004). High-sensitivity immunofluorescence/flow cytometry: detection of cytokine receptors and other low-abundance membrane molecules. *Current Protocols in Cytometry*.

[13] Arce M. A., Thompson E. S., Wagner S., Coyne K. E., Ferdman B. A., Lublin D. M. (1993). Molecular cloning of RhD cDNA derived from a gene present in RhD-positive, but not RhD-negative individuals. *Blood*.

